# Exploring the antifungal potential of *Cannabis sativa*-derived stilbenoids and cannabinoids against novel targets through *in silico* protein interaction profiling

**DOI:** 10.3389/fchem.2024.1515424

**Published:** 2025-01-06

**Authors:** Kevser Kübra Kırboğa, Aman Karim, Ecir Uğur Küçüksille, Mithun Rudrapal, Johra Khan, Raghu Ram Achar, Ekaterina Silina, Natalia Manturova, Victor Stupin

**Affiliations:** ^1^ Faculty of Engineering, Department of Bioengineering, Bilecik Şeyh Edebali University, Bilecik, Türkiye; ^2^ Faculty of Multidisciplinary Studies, Department of Biological Sciences, National University of Medical Sciences, Rawalpindi, Pakistan; ^3^ Faculty of Engineering, Department of Computer Engineering, Isparta Suleyman Demirel University, Isparta, Türkiye; ^4^ Department of Pharmaceutical Sciences, School of Biotechnology and Pharmaceutical Sciences, Vignan’s Foundation for Science, Technology and Research, Guntur, India; ^5^ Department of Medical Laboratory Sciences, College of Applied Medical Sciences, Majmaah University, Al Majmaah, Saudi Arabia; ^6^ Division of Biochemistry, School of Life Sciences, JSS Academy of Higher Education and Research, Mysuru, India; ^7^ Institute of Digital Biodesign and Modeling of Living Systems, I. M. Sechenov First Moscow State Medical University (Sechenov University), Moscow, Russia; ^8^ Department of Surgery, Pirogov Russian National Research Medical University, Moscow, Russia

**Keywords:** cannabinoids, stilbenoids, antifungal agents, molecular docking, molecular dynamics simulation, *Cannabis sativa*

## Abstract

Cannabinoid and stilbenoid compounds derived from *Cannabis sativa* were screened against eight specific fungal protein targets to identify potential antifungal agents. The proteins investigated included Glycosylphosphatidylinositol (GPI), Enolase, Mannitol-2-dehydrogenase, GMP synthase, Dihydroorotate dehydrogenase (DHODH), Heat shock protein 90 homolog (Hsp90), Chitin Synthase 2 (CaChs2), and Mannitol-1-phosphate 5-dehydrogenase (M1P5DH), all of which play crucial roles in fungal survival and pathogenicity. This research evaluates the binding affinities and interaction profiles of selected cannabinoids and stilbenoids with these eight proteins using molecular docking and molecular dynamics simulations. The ligands with the highest binding affinities were identified, and their pharmacokinetic profiles were analyzed using ADMET analysis. The results indicate that GMP synthase exhibited the highest binding affinity with Cannabistilbene I (−9.1 kcal/mol), suggesting hydrophobic solid interactions and multiple hydrogen bonds. Similarly, Chitin Synthase 2 demonstrated significant binding with Cannabistilbene I (−9.1 kcal/mol). In contrast, ligands such as Cannabinolic acid and 8-hydroxycannabinolic acid exhibited moderate binding affinities, underscoring the variability in interaction strengths among different proteins. Despite promising *in silico* results, experimental validation is necessary to confirm therapeutic potential. This research lays a crucial foundation for future studies, emphasizing the importance of evaluating binding affinities, pharmacokinetic properties, and multi-target interactions to identify promising antifungal agents.

## Graphical Abstract

**Figure:**
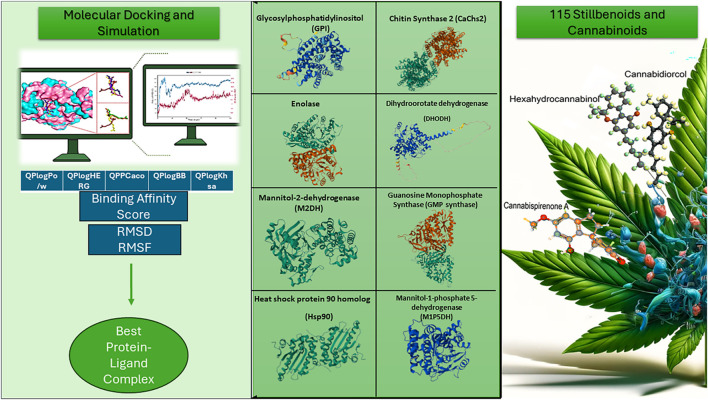
The objective of this study is to identify novel antifungal agents by exploring the interactions between cannabinoids and stilbenoids derived from Cannabis sativa and eight fungal protein targets that play critical roles in fungal pathogenicity and survival. These proteins were chosen based on their essential functions of cell wall integrity, metabolic processes, stress response, and other vital cellular activities essential for the proliferation and virulence of fungal pathogens. Targeting multiple pathways aims to evaluate whether these natural compounds can effectively inhibit crucial mechanisms in fungal cells, thereby offering a potential strategy to combat antifungal resistance. Through molecular docking and dynamics simulations, this study seeks to determine these compounds' specific binding affinities, stability, and pharmacokinetic properties, laying the groundwork for future experimental validation and potential therapeutic development.

## 1 Introduction

The increasing prevalence of fungal infections and antifungal-resistant strains necessitates discovering new and effective antifungal agents. *Cannabis sativa* has high therapeutic potential due to its diverse biologically active compounds (Clarke and Merlin, 2015). Stilbenoids and cannabinoids are significant compounds derived from *Cannabis sativa* that exhibit various biological activities. Previous studies have highlighted their anti-inflammatory, anticancer, and antibacterial properties (Glodowska, 2016; Hanane and Mohammed, 2021; Hourfane et al., 2023; Reiss, 2010; Sun, 2023). Research on the effects of stilbenoids and cannabinoids in different biological systems has shown that these compounds can be used in a broad therapeutic spectrum (Jeong et al., 2019). However, studies investigating the interactions of stilbenoids and cannabinoids with fungal proteins and the antifungal efficacy of these interactions are limited.

Recent studies have explored the potential of natural metabolites derived from plants and fungi for antiviral, antidiabetic, and receptor-targeted purposes using *in silico* techniques. For example, Khan et al. conducted a molecular docking (MD) and dynamics simulation (MDS) study on secondary metabolites derived from medicinal fungi as potential inhibitors for COVID-19 treatment. This study highlighted several compounds that effectively targeted viral proteins such as the main protease and TMPRSS2, demonstrating the efficacy of fungi-derived natural products in combating viral infections (Khan et al., 2023). Additionally, another study investigated *Cannabis* constituents as potential candidates against diabetes mellitus using MD, dynamics simulations, and ADMET investigations, showing promising therapeutic potential (Abchir et al., 2023). Moreover, a recent study by Aissaoui et al. explored the anticancer potential of cannabidiol using computational methods, including MD, which are similar to the methodologies applied in this study. Their results indicated significant binding energies and interactions with targeted proteins, suggesting that cannabidiol could be synthesized and tested as a potential treatment for various types of human cancer (Aissaoui et al., 2024). Furthermore, virtual screening of cannabinoid analogs against CB1 and CB2 receptors using MD and MDS has also shown that these compounds interact significantly with key targets associated with these receptors, underscoring the therapeutic potential of cannabinoids beyond their known uses (Aviz-Amador et al., 2021).

This study aims to identify antifungal compounds by screening stilbenoids and cannabinoids reported from *Cannabis sativa* against new protein targets. A comprehensive literature review indicates a lack of extensive studies on the interactions of stilbenoids and cannabinoids with fungal proteins and their antifungal potential.

In this study, cannabinoid and stilbenoid compounds derived from *Cannabis sativa* were selected considering their structural diversity, biological activities and documented antifungal potential. Cannabiorcol (C₁₇H₁₈O₂) was included due to its unique structure and antimicrobial activities against various microorganisms. In the literature, it has been reported that this compound binds to the fungal cell membrane, destabilizes the cell wall and inhibits the growth of pathogenic cells (Appendino et al., 2008). Δ9-trans-Tetrahydrocannabinol (Δ9-trans-THC) (C₁₇H₂₂O₂) is an isomer of tetrahydrocannabinol (THC) and can establish strong interactions with cellular membranes due to its lipophilic properties. This compound was included in the study due to its antifungal potential, considering the known antimicrobial effects of THC (Kogan and Mechoulam, 2007). Cannabidiol (C₁₇H₂₂O₂) is a Cannabidiol (CBD) analog that exhibits potent antibiofilm activity against fungal species such as *Candida* albicans, offering the potential to target biofilm-based resistance mechanisms of pathogens (Feldman et al., 2021). CBD was selected in our study to evaluate its potential interactions with antifungal target proteins. In the literature, CBD has been shown to exhibit strong anti-inflammatory effects in human keratinocyte (HaCaT) cells stimulated with polyinosinic-polycytidylic acid [poly-(I:C)]. CBD suppresses the production of inflammatory markers such as monocyte chemotactic protein-2 (MCP-2), interleukin-6 (IL-6), interleukin-8 (IL-8), and tumor necrosis factor-α (TNF-α) in these cells in a dose-dependent manner (Petrosino et al., 2018). These effects have been shown to occur by activating cannabinoid type-2 (CB2) and transient receptor potential vanilloid type-1 (TRPV1) receptors. In addition, the absence of cytotoxic effects of CBD suggests that it can be considered a safe anti-inflammatory agent. CBD is predicted to have the potential to regulate cytokine and chemokine production during fungal infections. These properties make CBD a candidate that may support multiple binding mechanisms with antifungal target proteins and warrants comprehensive investigation in our study (Petrosino et al., 2018).

The pharmacokinetic properties of stilbenoids were decisive in the selection of certain compounds in our study. For example, Cannabistilbene I was a strong candidate, exhibiting properties such as high permeability through human skin (32.755 cm/s × 10⁷), jejunal permeability (5.229 cm/s × 10⁴) and high penetration into the blood-brain barrier (BBB) (81% reliability). Canniprene and 3,4′-Dihydroxy-5,3′-dimethoxy-5′-isoprenylbibenzyl fully comply with Lipinski’s “Rule of 5” criteria (0 violations) and have an effective pharmacokinetic profile in terms of both skin and intestinal permeability. The high BBB penetration of stilbenoids (80%–96%) and their potential to bind to antifungal target proteins are consistent with their biological activities. In particular, it is anticipated that these compounds will support multiple target mechanisms with their capacity to form hydrogen bonds, lipophilic profiles and antifungal proteins (O'Croinin et al., 2023).

In the selection of stilbenoids, their strong antioxidant properties and cell membrane destabilization potential were taken into consideration. Cannabidivarin (CBDV) was selected in our study to evaluate its potential interactions with antifungal target proteins. In the literature, CBDV has been reported to be a non-psychoactive phytocannabinoid and to have both activation and desensitization effects on the TRPA1 (Transient Receptor Potential Ankyrin 1) channel family. This feature reveals its capacity to regulate inflammation-related parameters, especially cytokine production and intestinal permeability (Pagano et al., 2019). In studies conducted in mice, CBDV has been shown to reduce neutrophil infiltration, correct imbalances in the intestinal microbiota, and suppress the expression of inflammatory cytokines (IL-1β, IL-6, MCP-1). In addition, the ability of CBDV to reduce cytokine expression has been reported in biopsies taken from pediatric patients with active ulcerative colitis (UC). It is predicted that this compound may also show similar interactions with fungal proteins through mechanisms related to the control of inflammatory processes at the cellular level. CBDV, which is considered a safe phytocannabinoid in humans, was included in our study as a strong candidate for the evaluation of interactions with antifungal target proteins due to both its biological activity and pharmacokinetic profile (Pagano et al., 2019). These compounds were evaluated in the study due to their chemical diversity and potential for developing multi-target antifungal agents. In particular, their hydrogen bonding capacity (O'Croinin et al., 2023), lipophilic profiles (Akinwumi et al., 2018; Castellano et al., 2014; Lucas et al., 2018) and binding compatibility (Platella et al., 2021) with target proteins (Wahedi et al., 2020) constituted an important part of the selection criteria.

Accordingly, this research evaluates the interactions between eight proteins, critical in fungal pathogenicity, and specific stilbenoids and cannabinoids. The selected proteins include Glycosylphosphatidylinositol (GPI) from *Candida albicans*, which plays a vital role in cell wall structure and function. Inhibition of GPI proteins can disrupt fungal cell wall synthesis and reduce virulence (Chaffin, 2008). Enolase (AfEno1) from *Aspergillus fumigatus* is crucial in glycolysis and energy metabolism of pathogenic fungi, with inhibition shown to halt fungal growth (Dasari et al., 2019). Mannitol-2-dehydrogenase (M2DH) from Aspergillus fumigatus is involved in mannitol metabolism and osmoregulation, where inhibition increases sensitivity to osmotic stress (Suvarna et al., 2000). Guanosine Monophosphate Synthase (GMP synthase) from *A. fumigatus* is essential in guanine nucleotide synthesis, and its inhibition can impede cell division and pathogenicity (Kingsbury and McCusker, 2010). DHODH from *Aspergillus nidulans* is crucial in pyrimidine biosynthesis, with inhibition shown to stop fungal growth effectively (Nara et al., 2000). Heat shock protein 90 homolog (Hsp90) from *Candida* albicans assists in protein folding and stress response, with inhibition disrupting pathogenicity (Cowen and Lindquist, 2005). Chitin Synthase 2 (CaChs2) from *C. albicans* plays a role in chitin synthesis, with inhibition leading to weakened cell walls (Munro and Gow, 2001). Mannitol-1-phosphate 5-dehydrogenase (M1P5DH) from *Neosartorya fumigata* is involved in mannitol metabolism, where inhibition affects osmoregulation and survival (Ruijter et al., 2004). These proteins are critical in fungal cells' survival and pathogenic mechanisms, making them suitable targets. These eight specific proteins were selected based on their crucial roles in fungal cell survival, pathogenicity, and resistance mechanisms. Each protein represents a key biological pathway or structural component essential for fungal virulence and survival, such as cell wall synthesis, energy metabolism, stress response, and nucleotide synthesis. Targeting multiple vital pathways simultaneously increases the likelihood of identifying potential antifungal agents that can overcome existing drug resistance mechanisms. The inhibition of these proteins involved in diverse but essential functions provides a broad spectrum approach to compromising fungal pathogenicity and highlights their significance as promising antifungal drug targets. Docking studies were conducted using PyRx software and the AutoDock Vina algorithm, followed by advanced MDS with Schrödinger Maestro 2021–3 software and the Desmond module. Simulation data were analyzed to evaluate protein-ligand interactions, binding energies, and stabilities. Additionally, ADMET analysis was performed using the Schrödinger QikProp module to assess the drug-like properties of the compounds. The novelty of this study lies in its comprehensive *in silico* approach to identify potential antifungal agents among stilbenoids and cannabinoids, focusing on eight essential fungal proteins that play key roles in pathogenicity. Unlike previous studies, which largely focused on these compounds' anti-inflammatory or anticancer properties, this research explores the antifungal potential by systematically evaluating binding affinities, dynamic behaviors, and pharmacokinetic properties. This integrated approach provides a novel insight into the potential of *Cannabis sativa*-derived compounds as antifungal agents, offering a foundation for future *in-vitro* and *in-vivo* validations. Ultimately, this study aims to contribute to developing new therapeutic strategies targeting antifungal resistance, an emerging global health challenge.

## 2 Materials and methods

The primary objective of this study is to investigate the potential antifungal compounds by screening stilbenoid and cannabinoid compounds derived from *Cannabis sativa* against novel protein targets. To this end, MD analyses were conducted utilizing PyRx software (Dallakyan and Olson, 2015). Furthermore, the assessment of drug-like properties, encompassing absorption, distribution, metabolism, excretion, and toxicity (ADMET), alongside MDS, was performed using Schrödinger software (Schrödinger, 2016). These extensive analyses have provided a deeper insight into the efficacy and safety profiles of the investigated compounds.

### 2.1 Data collection

This study investigates the antifungal potential of stilbenoid and cannabinoid compounds derived from Cannabis sativa, utilizing eight fungal protein targets (Table 1; Table 2, and Supplementary Data S1). Eight fungal proteins selected in this study were identified due to their critical roles in the survival and pathogenicity mechanisms of fungal pathogens and were used as primary targets to evaluate the efficacy of antifungal compounds. GPI protein plays a central role in cell wall biosynthesis, ensuring fungal cell stability; targeting this protein allowed weakening of cellular structural integrity (Samalova et al., 2020). Enolase is a key enzyme in glycolysis and energy metabolism and has been evaluated as a potential target for inhibition of energy production (Avilán et al., 2011; Langenhorst et al., 2023). M2DH and M1P5DH proteins play an important role in the adaptation of fungal cells to stress conditions by participating in osmoregulation and mannitol metabolism; inhibition of these proteins contributed to the disruption of osmotic balance (Meena et al., 2015). GMP synthase (Rodriguez-Suarez et al., 2007) ve DHODH (Zameitat et al., 2007) proteins are essential for genetic material production and cell division by participating in purine and pyrimidine biosynthesis, respectively; targeting these proteins has demonstrated the potential to limit fungal growth by interfering with DNA/RNA synthesis. Heat Shock Protein 90 (Hsp90), a chaperone protein that regulates stress responses and protein folding processes, has been targeted to reduce adaptability to environmental stresses (Gupta, 1995). CaChs2 protein has a critical role in the synthesis of chitin in the cell wall and has been used as an important target in weakening cellular mechanical stability (Banks et al., 2005). These proteins represent vital fungal processes such as energy metabolism, cell wall stability, genetic material synthesis and osmoregulation and have made significant contributions to the main aim of the study to develop multi-target antifungal strategies and overcome antifungal resistance mechanisms.

**TABLE 1:** Proteins and their sources were used in this study.

Protein name	Source database	Source organism	Reference
Glycosylphosphatidylinositol (GPI)	AlphaFold	Candida albicans	UniProt ID: Q873N2
Enolase (AfEno1)	RCSB PDB	Aspergillus fumigatus	7rhv.pdb
Mannitol-2-dehydrogenase (M2DH) (AfM1PDH)	RCSB PDB	Aspergillus fumigatus	7rk4.pdb
Guanosine Monophosphate Synthase (GMP synthase)	RCSB PDB	Aspergillus fumigatus	7mo6.pdb
Dihydroorotate dehydrogenase (DHODH)	AlphaFold	Aspergillus nidulans	UniProt ID: Q12610
Heat shock protein 90 homolog (Hsp90)	RCSB PDB	Candida albicans	6cjs.pdb
Chitin Synthase 2 (CaChs2)	RCSB PDB	*Candida* albicans	7STO.pdb
Mannitol-1-phosphate 5-dehydrogenase (M1P5DH) AfM2DH	AlphaFold	Neosartorya fumigata	UniProt ID: A0A222WJM7

**TABLE 2:** List of Cannabinoid and Stilbenoid compounds analyzed in this study. The continuation of the list can be found in the supplementary materials.

Compound type	Name of compound	Molecular formula
Cannabinoid	Cannabiorcol	C17H18O2
Cannabinoid	Δ9-trans-Tetrahydrocannabiorcol	C17H22O2
Cannabinoid	Cannabidiorcol	C17H22O2
Cannabinoid	Nor-Cannabivarin (cannabinol-C2)	C18H20O2
Cannabinoid	Δ9-trans-Tetrahydrocannabiorcolic acid	C18H22O4
Stilbenoid	Cannabispirone	C15H18O3
Stilbenoid	Cannabispirenone-A	C15H16O3
Stilbenoid	Isocannabispirenone	C15H20O3
Stilbenoid	Isocannabispiradienone	C15H14O3
Stilbenoid	Cannabispirenone-B	C15H16O3

While most of the analyzed targets had experimentally resolved structures available in the RCSB PDB database (Berman et al., 2000), two of the proteins lacked structural data. For these two proteins, AlphaFold’s state-of-the-art protein structure prediction algorithm (Varadi et al., 2021) was employed to generate high-confidence models. This integration of AlphaFold predictions addressed a critical gap by enabling the inclusion of these fungal proteins, significantly enhancing the study’s comprehensiveness. Structural validation showed that AlphaFold-predicted models aligned well with similar proteins from the PDB, as indicated by low RMSD values, ensuring the reliability of the predicted structures. Additionally, these models revealed critical binding sites and conformational details that facilitated robust molecular docking and dynamics simulations. By incorporating AlphaFold predictions, this study broadened the target protein repertoire, allowing for a more complete assessment of the antifungal potential of the tested compounds. The use of AlphaFold not only supplemented missing structural data but also enabled comparative analyses with human homologs, aiding in the identification of selective antifungal targets (Varadi and Velankar, 2022). While AlphaFold models require experimental validation (Mirabello et al., 2024), their inclusion provided a strong foundation for hypothesis-driven research and highlighted the transformative potential of this tool in drug discovery for less-characterized fungal targets. The comprehensive integration of AlphaFold-predicted models not only enhanced the study’s ability to explore diverse fungal protein structures but also provided a robust framework for evaluating their critical roles in pathogenicity and survival.

### 2.2 Biological roles and signaling pathways of eight proteins

In this study, Cytoscape (v. 3.10.3) was used for network-based visualization and analysis together with KEGG Pathway (Varadi and Velankar, 2022), UniProt (Coudert et al., 2022) and Reactome (Milacic et al., 2023) databases to determine the molecular and cellular functions of eight fungal protein targets and to analyze the signaling pathways associated with these proteins. The roles of proteins in biological processes, molecular functions and cellular components were analyzed based on the information obtained from these databases. First, the functional properties and associated biological processes of each protein were characterized in detail using the UniProt database. The KEGG Pathway database was used to identify and detail the metabolic pathways and possible signaling mechanisms in which these proteins are involved. The Reactome database was used for in-depth analysis of specific biological pathways in which the proteins are involved, and a comprehensive and systematic evaluation of these pathways was performed.

In addition, Cytoscape software (Shannon et al., 2003) was used to visualize the obtained data relationships and interactions between proteins more clearly. This software was used as a critical tool in detailing the relationships and common functional pathways of proteins, providing the opportunity to effectively visualize the connections and shared biological processes between proteins. These comprehensive database searches and network analyses provided information about the cellular locations and molecular functions of the proteins, as well as revealed their interrelationships and common functional pathways. This methodological approach allowed for extensive functional analysis to understand both independent and coordinated roles of the proteins and provided a multifaceted perspective to evaluate the antifungal therapeutic potential of related proteins.

### 2.3 Molecular docking

Docking studies were performed using the PyRx software, incorporating the AutoDock Vina algorithm (Morris et al., 2009). Before docking, proteins were prepared by removing water molecules and cofactors, followed by energy minimization to optimize their structure. Ligands were retrieved from chemical databases in SMILES format, and their initial structures were subjected to a systematic workflow for generating conformations. Using Open Babel’s systematic rotor search method, multiple ligand conformations were generated by exploring rotatable bonds to ensure a comprehensive sampling of conformational space, vital for accurately representing biologically relevant poses. The best conformer based on energy minimization was selected for docking. For each protein, a grid box around the active site was defined, with dimensions set to adequately encompass the binding region (x = 40 Å, y = 40 Å, z = 40 Å). The docking procedure was carried out using the AutoDock Vina algorithm, with specific center coordinates and grid dimensions settings. Parameters such as exhaustiveness (set at 8) and the number of binding modes to be analyzed (set at 10) were adjusted to ensure thorough exploration of potential ligand orientations. The outcomes of the docking process, including binding affinity values and binding positions, were carefully evaluated to identify the most promising candidates. Ligands showing the highest binding affinities were subsequently selected for further MDS.

### 2.4 Molecular dynamics simulations (MDS)

In this study, MDS was conducted on eight target proteins, each paired with two ligands, to evaluate each protein-ligand complex’s binding stability and interaction profiles. The simulations were performed using the Schrödinger Maestro 2021–3 software suite for 100 nanoseconds (ns) at 310 K (K) temperature within the NPT ensemble. Schrödinger’s Desmond module facilitated the simulations, allowing for the analysis of structural stability, dynamic behavior, and specific interactions at the protein-ligand binding sites.

The selected protein-ligand complexes underwent solvation and ionization processes following the initial docking. The TIP3P water model was used for solvation, and the systems were neutralized with Na+ and Cl-ions to maintain physiological conditions. The simulation protocol consisted of three main phases: a heating phase from 0 to 10 ns, an equilibration phase from 10 to 20 ns, and a production phase from 20 to 100 ns. Data from the simulations were analyzed to calculate Root Mean Square Deviation (RMSD) and Root Mean Square Fluctuation (RMSF) values, providing insights into protein-ligand interactions, binding energies, and overall system stability.

Further analysis was conducted using Schrödinger’s Prime MM-GBSA and Maestro modules to calculate the binding free energy and to characterize ligand binding modes. The force field parameters for the small molecules were generated using the OPLS3e force field within the Schrödinger software. This parameterization utilized the molecular structures of the ligands, and the automatic force field assignment ensured an accurate representation of intramolecular and intermolecular interactions. These comprehensive analyses provided profound insights into the protein-ligand complexes' interaction profiles and binding stabilities.

### 2.5 ADMET analysis

The ADMET (Absorption, Distribution, Metabolism, Excretion, and Toxicity) analysis was performed using the Schrödinger QikProp module to assess the drug-like properties of the selected compounds. This analysis aimed to evaluate various pharmacokinetic parameters crucial for determining the safety and efficacy of potential drug candidates. Specifically, QikProp was used to calculate parameters such as molecular weight (mol_MW), solvent-accessible surface area (SASA), the number of hydrogen bond donors (donorHB) and acceptors (accptHB), logP (partition coefficient), potential for hERG inhibition (QPlogHERG), Caco-2 cell permeability (QPPCaco), BBB permeability (QPlogBB), and plasma protein binding affinity (QPlogKhsa). These pharmacokinetic descriptors comprehensively evaluated the compounds' ADMET profiles, allowing for an informed assessment of their drug-likeness, bioavailability, and potential safety profiles. In addition to pharmacokinetic evaluations, toxicity analyses were also performed using the Schrödinger QikProp module to provide safety profiles of the tested compounds. QikProp provided reliable estimates of toxicity-related parameters such as Ames test results (mutagenicity potential), LD50 values (acute oral toxicity, in mg/kg), and cardiotoxicity potential via hERG channel inhibition (QPlogHERG). These parameters were calculated together with other descriptors such as accessible surface area in solution (SASA), logP (partition coefficient), and hydrogen bonding capacity (donorHB and accptHB), providing a comprehensive understanding of the safety profile of each compound. While Ames test estimates were classified as mutagenic or non-mutagenic, LD50 values provided quantitative information on the risk of acute toxicity. The integration of these toxicity descriptors with pharmacokinetic properties provided a balanced assessment of the therapeutic potential of compounds and allowed informed decisions to be made during the antifungal drug development process.

## 3 Results

### 3.1 Biological roles and signaling pathways

**FIGURE 1:**
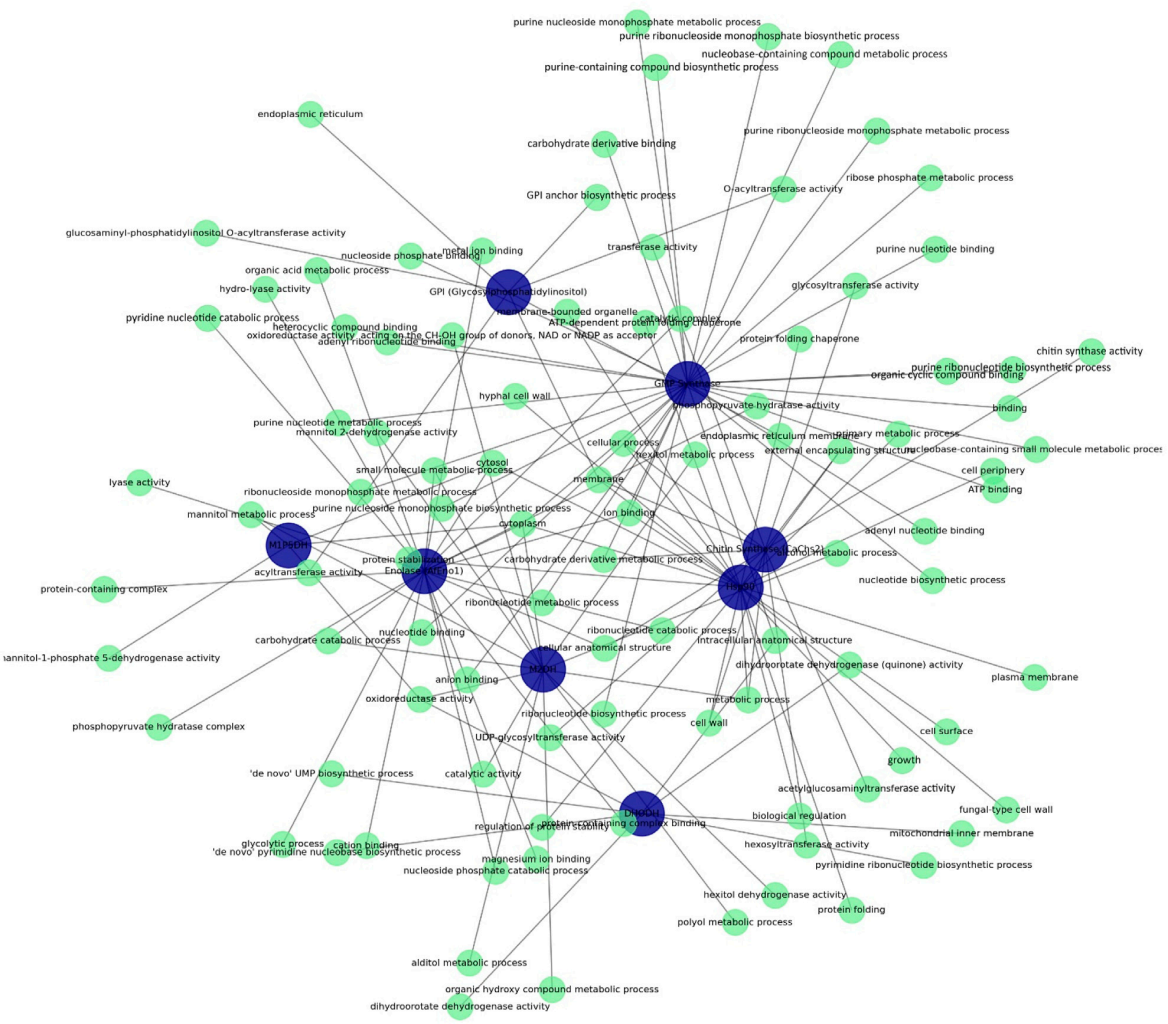
Functional interaction network of target proteins highlighting molecular functions, biological processes, and cellular components.

This study provides a comprehensive evaluation of the functional roles and involvement of eight different fungal target proteins in biological processes (Table 3; Figure 1). GPI (Glycosylphosphatidylinositol) is localized in the endoplasmic reticulum and membrane, playing a central role in GPI anchor biosynthesis, which is critical for regulating protein attachment to the cell surface. Enolase is found in the cytoplasm and cytosol, contributing to fundamental energy metabolism pathways, specifically glycolysis and ribonucleotide catabolism. M2DH exhibits oxidoreductase activity involved in the metabolism of mannitol and other polyol derivatives, supporting energy storage and osmotic balance. GMP Synthase plays a key role in nucleotide metabolic pathways, such as ribonucleotide and purine biosynthesis, while also contributing to cellular biosynthesis through diverse molecular activities like ATP binding. DHODH localizes the mitochondrial inner membrane, where it facilitates *de novo* pyrimidine biosynthesis, which is crucial for nucleotide production. Hsp90 plays a role in protein folding, stabilization, and biological regulation processes, regulating stress responses and cellular growth. CaChs2 is involved in chitin biosynthesis and glycosyltransferase activities, participating in cell wall biosynthesis and maintaining cellular stability. M1P5DH is involved in mannitol metabolism and operates in the cytoplasm to regulate energy storage and osmotic balance.

**TABLE 3:** Comprehensive representation of the molecular functions, biological processes, and cellular localizations of eight target proteins.

Go category	GPI (glycosylphosphatidylinositol)	Enolase (AfEno1)	M2DH	GMP synthase	DHODH	Hsp90	Chitin synthase (CaChs2)	M1P5DH
Molecular Function	O-acyltransferase activity, acyltransferase activity, glucosaminyl-phosphatidylinositol O-acyltransferase activity	cation binding, magnesium ion binding, ion binding, metal ion binding, lyase activity, catalytic activity, protein-containing complex binding, hydro-lyase activity, phosphopyruvate hydratase activity	catalytic activity, oxidoreductase activity, mannitol 2-dehydrogenase activity, oxidoreductase activity acting on the CH-OH group of donors, hexitol dehydrogenase activity	ion binding, binding, nucleoside phosphate binding, purine nucleotide binding, organic cyclic compound binding, adenyl nucleotide binding, nucleotide binding, heterocyclic compound binding, ATP binding, carbohydrate derivative binding, anion binding	oxidoreductase activity, dihydroorotate dehydrogenase activity, dihydroorotate dehydrogenase (quinone) activity	ion binding, ATP-dependent protein folding chaperone, protein folding chaperone	UDP-glycosyltransferase activity, glycosyltransferase activity, chitin synthase activity, acetylglucosaminyltransferase activity, transferase activity, hexosyltransferase activity	oxidoreductase activity, mannitol-1-phosphate 5-dehydrogenase activity
Biological Process	GPI anchor biosynthetic process	glycolytic process, ribonucleotide catabolic process, carbohydrate catabolic process, pyridine nucleotide catabolic process, small molecule metabolic process, organic acid metabolic process, nucleoside phosphate catabolic process	carbohydrate catabolic process, small molecule metabolic process, mannitol metabolic process, hexitol metabolic process, polyol metabolic process, organic hydroxy compound metabolic process, alcohol metabolic process, alditol metabolic process, metabolic process	carbohydrate derivative metabolic process, ribose phosphate metabolic process, purine ribonucleoside monophosphate biosynthetic process, nucleotide biosynthetic process, purine ribonucleotide biosynthetic process, nucleobase-containing compound metabolic process, ribonucleotide biosynthetic process, purine nucleoside monophosphate biosynthetic process, purine ribonucleoside monophosphate metabolic process, purine nucleotide metabolic process, ribonucleotide metabolic process, ribonucleoside monophosphate metabolic process, nucleobase-containing small molecule metabolic process, purine-containing compound biosynthetic process	'*de novo*' pyrimidine nucleobase biosynthetic process, pyrimidine ribonucleotide biosynthetic process, ‘*de novo*' UMP biosynthetic process	primary metabolic process, metabolic process, cellular process, regulation of protein stability, protein stabilization, protein folding, biological regulation, growth	carbohydrate derivative metabolic process, metabolic process, cellular process	mannitol metabolic process
Cellular Component	endoplasmic reticulum, endoplasmic reticulum membrane, membrane	cytoplasm, catalytic complex, cytosol, phosphopyruvate hydratase complex, protein-containing complex, cellular anatomical structure	cytoplasm	cytoplasm, intracellular anatomical structure	mitochondrial inner membrane, intracellular anatomical structure	membrane, cytoplasm, cytosol, plasma membrane, fungal-type cell wall, cell wall, external encapsulating structure, hyphal cell wall, cell periphery, cell surface, membrane-bounded organelle	membrane, cell wall, external encapsulating structure, cellular anatomical structure	cytoplasm, cytosol

The analysis of shared biological processes and cellular components highlights how these proteins interact with each other and the potential synergistic relationships among them (Supplementary Data S2). For instance, Enolase and GMP Synthase share similar molecular functions, such as “ATP binding” and “organic compound binding,” indicating parallel roles in energy metabolism. Additionally, GMP Synthase and Hsp90 both localize in the cytosol and plasma membrane, whereas CaChs2 and GPI contribute to cell wall biosynthesis, maintaining cellular stability and regulating membrane-associated structural processes. This comprehensive assessment underscores the coordinated roles of these eight fungal proteins in biological processes and highlights their importance as potential targets for antifungal strategies.

### 3.2 Molecular docking

As a result of the MD and MDS, the ligands with the best binding affinities and their pharmacokinetic profiles were evaluated. According to Table 4, the docking studies reveal the binding affinities between various proteins and selected cannabinoid and stilbenoid compounds, measured in kcal/mol. Using PyRx software and the AutoDock Vina (Morris et al., 2009) algorithm, we assessed the interaction strengths of these protein-ligand pairs. The proteins included Hsp90, GMP synthase, Enolase, Mannitol-2-dehydrogenase, CaChs2, Glycosylphosphatidylinositol, M1P5DH, and DHODH. For Hsp90, the binding affinities with Hexahydrocannabinol and 5-OH-7-MeO-Indan-Spiro-Cyclohexane were −8.2 and −8.3 kcal/mol, respectively. GMP synthase showed a high binding affinity with Cannabistilbene I at −9.1 kcal/mol and a lower affinity with Δ9-cis-THC at −7.2 kcal/mol. Enolase had binding affinities of −7.1 kcal/mol with Cannabinolic acid and −7.8 kcal/mol with 8-hydroxycannabinolic acid. Mannitol-2-dehydrogenase interacted with Δ9-trans-THC and 8a-Hydroxy-Δ9-trans-THC showing affinities of −7.1 and −7.7 kcal/mol, respectively. CaChs2 strongly binds with Cannabistilbene I at −9.1 kcal/mol and moderate binding with Cannabidivarinic acid (CBDVA) at −7.6 kcal/mol. Glycosylphosphatidylinositol had affinities of −8.2 kcal/mol with both 3-Butyl-Δ9-THC and Δ9-trans-THC. M1P5DH showed affinities of −7.1 kcal/mol with Cannabinolic acid and −7.8 kcal/mol with 8-hydroxycannabinolic acid. Lastly, DHODH exhibited affinities of −7.6 kcal/mol with Cannabiglendol and −7.5 kcal/mol with Cannabicitran. The highest binding affinities were observed with GMP synthase and CaChs2 interacting with Cannabistilbene I, both at −9.1 kcal/mol, indicating a strong interaction. In comparison, the lowest affinity values were noted at −7.1 kcal/mol for several protein-ligand pairs, suggesting moderate interactions. These results highlight the potential of these compounds as antifungal agents and provide a basis for further MDS and biological validations.

**TABLE 4:** Docking binding affinities, MM-GBSA binding free energies, and ADMET properties of protein-ligand complexes.

Protein	Ligand	Docking binding affinity (kcal/mol)	MM-GBSA ΔG (kcal/mol)	QPlogPo/w (lipophilicity)	QPlogHERG (hERG inhibition, log (mol/L))	QPPCaco (intestinal permeability, nm/s)	Ames test (mutagenicity)	LD50 (mg/kg)
Hsp90	Hexahydrocannabinol	−8.2	−82	5.724	−4.8	4,522.262	Non-Mutagenic	2000
Hsp90	5-OH-7-MeO-Indan-Spiro-Cyclohexane	−8.3	−83	3.45	−3.751	3,035.311	Non-Mutagenic	1500
GMP synthase	Cannabistilbene I	−9.1	−91	4.004	−3.435	1,318	Non-Mutagenic	2500
GMP synthase	Δ9-cis-Tetrahydrocannabinol	−7.2	−72	5.663	−4.894	4,571.459	Mutagenic	1000
Enolase	Cannabinolic acid	−7.1	−71	5.533	−3.228	276.554	Non-Mutagenic	2000
Enolase	8-hydroxycannabinolic acid	−7.8	−78	4.8	−3.197	95.563	Non-Mutagenic	1900
Mannitol-2-dehydrogenase	Δ9-trans-Tetrahydrocannabinol	−7.1	−71	5.466	−2.874	267.368	Non-Mutagenic	1800
Mannitol-2-dehydrogenase	8a-Hydroxy-Δ9-Transtetrahydrocannabinol	−7.7	−77	4.584	−4.819	1,915.497	Mutagenic	1200
Chitin Synthase 2	Cannabistilbene I	−9.1	−91	4.004	−3.435	1,318	Non-Mutagenic	2500
Chitin Synthase 2	Cannabidivarinic acid	−7.6	−76	4.874	−2.417	253.686	Non-Mutagenic	2000
Glycosylphosphatidylinositol	Butyl-Δ9-THC	−8.2	−82	5.262	−4.687	4,438.445	Mutagenic	1200
Glycosylphosphatidylinositol	Δ9-trans-Tetrahydrocannabiorcol	−8.2	−82	4.107	−4.049	4,453.091	Non-Mutagenic	1500
Mannitol-1-phosphate 5-dehydrogenase	Cannabinolic acid	−7.1	−71	5.533	−3.228	276.554	Non-Mutagenic	2000
Mannitol-1-phosphate 5-dehydrogenase	8-hydroxycannabinolic acid	−7.8	−78	4.8	−3.197	95.563	Non-Mutagenic	1900
DHODH	Cannabiglendol	−7.6	−76	4.258	−4.009	3,007.355	Non-Mutagenic	1700
DHODH	Cannabicitran	−7.5	−75	4.973	−4.511	9,906.038	Non-Mutagenic	1600

**FIGURE 2:**
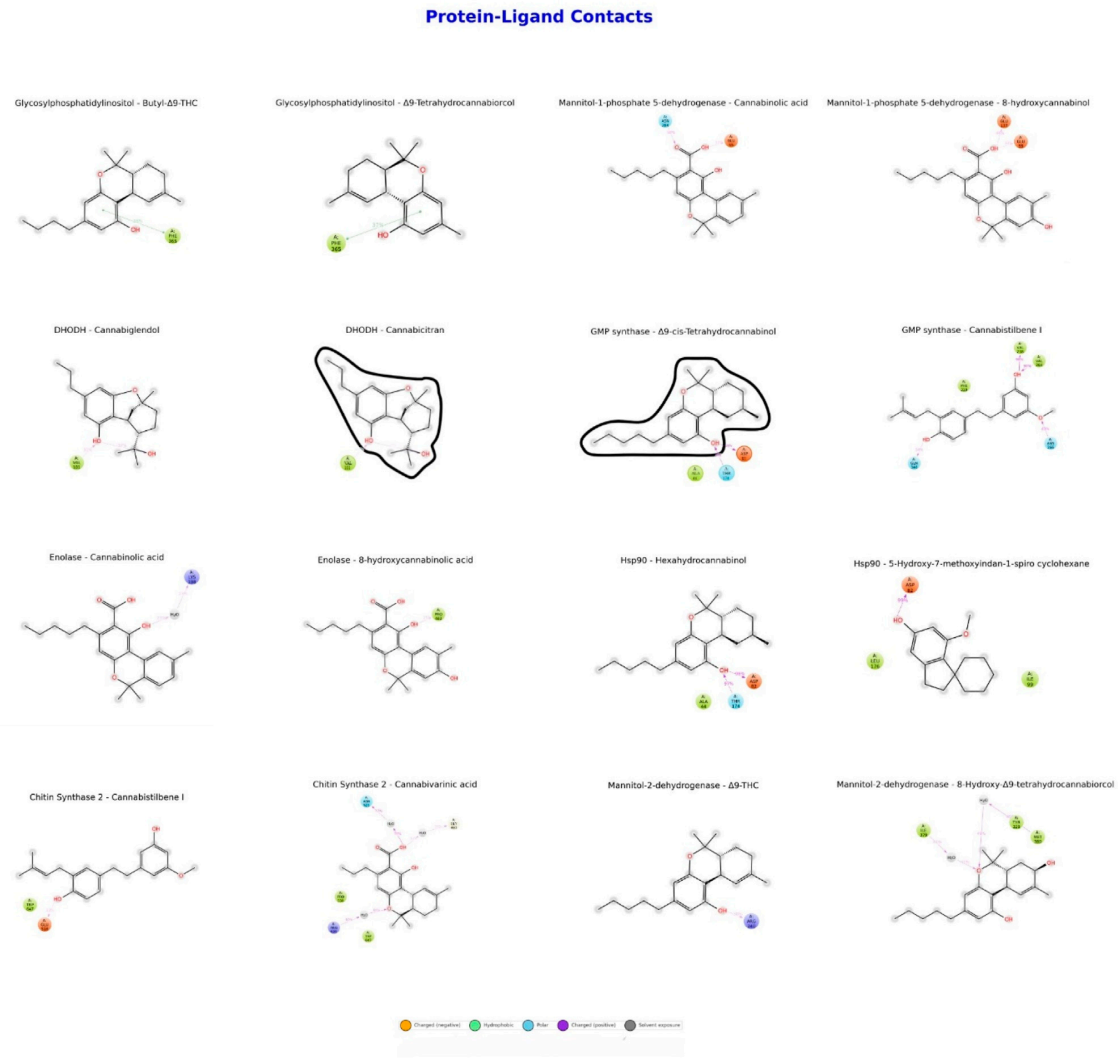
Binding affinities and interaction profiles of protein-ligand complexes.

As shown in Figure 2, the binding affinities and interaction profiles of eight different proteins with selected cannabinoid and stilbenoid compounds were evaluated. Hydrogen bonds and hydrophobic interactions play significant roles in the interactions of Hsp90 with Hexahydrocannabinol and 5-OH-7-MeO-Indan-Spiro-Cyclohexane. GMP synthase exhibited a high binding affinity with Cannabistilbene I at −9.1 kcal/mol, indicating strong hydrophobic interactions and multiple hydrogen bonds with the ligand. Significant contributions from hydrogen bonds and hydrophobic regions characterized enolase interactions with Cannabinolic acid and 8-hydroxycannabinolic acid. Mannitol-2-dehydrogenase demonstrates moderate binding affinities (−7.1 to −7.7 kcal/mol) with Δ9-trans-THC and 8a-Hydroxy-Δ9-trans-THC, where hydrogen bonds and hydrophobic interactions are crucial. CaChs2 strongly binds with Cannabistilbene I (−9.1 kcal/mol) but has a lower affinity with CBDVA (−7.6 kcal/mol). Glycosylphosphatidylinositol has binding affinities of −8.2 kcal/mol with both 3-Butyl-Δ9-THC and Δ9-trans-Tetrahydrocannabiorcol. M1P5DH exhibited binding affinities of −7.1 kcal/mol with Cannabinolic acid and −7.8 kcal/mol with 8-hydroxycannabinolic acid. Finally, DHODH showed binding affinities of −7.6 kcal/mol with Cannabiglendol and −7.5 kcal/mol with Cannabicitran. Solvent exposure is observed in the DHODH-Cannabicitran and GMP synthase-Δ9-cis-THC complexes.

### 3.3 ADMET profile prediction

Table 4 displays the ADMET properties of various ligands, highlighting significant differences in their pharmacokinetic and toxicity profiles. Hexahydrocannabinol shows high lipophilicity (QPlogPo/w 5.724) and potential HERG inhibition (−4.8), with excellent intestinal permeability (QPPCaco 4,522.262) but moderate brain penetration (QPlogBB −0.096). Its non-mutagenic classification and moderate LD50 value of 2000 mg/kg suggest an acceptable safety profile. In contrast, Cannabistilbene I, a standout candidate, exhibits moderate lipophilicity (QPlogPo/w 4.004) and HERG inhibition risk (−3.435), with lower intestinal permeability (QPPCaco 1,318) but strong toxicity results, including a non-mutagenic Ames test outcome and a favorable LD50 of 2,500 mg/kg, indicating low acute toxicity. Δ9-cis-THC and 3-Butyl-Δ9-THC both demonstrate high lipophilicity and HERG inhibition risk, coupled with mutagenic potential and lower LD50 values (1,000–1,200 mg/kg), raising safety concerns despite their substantial intestinal permeability.Enolase ligands, Cannabinolic acid, and 8-hydroxycannabinolic acid, display moderate lipophilicity, low intestinal permeability, and a non-mutagenic profile, with LD50 values of 2,000 mg/kg and 1,900 mg/kg, respectively, supporting their safety. DHODH ligands, Cannabiglendol and Cannabicitran, show moderate lipophilicity and HERG inhibition, with Cannabicitran standing out due to its notably higher intestinal permeability (QPPCaco 9,906.038) and an LD50 of 1,600 mg/kg, indicating potential safety concerns compared to Cannabiglendol (LD50 1,700 mg/kg).Overall, these results underline the balance between efficacy and safety, with Cannabistilbene I emerging as a promising candidate due to its favorable ADMET and toxicity profile, while compounds such as Δ9-cis-THC and 3-Butyl-Δ9-THC require caution due to their mutagenicity and lower LD50 values.

### 3.4 Molecular dynamics simulation analysis

**FIGURE 3:**
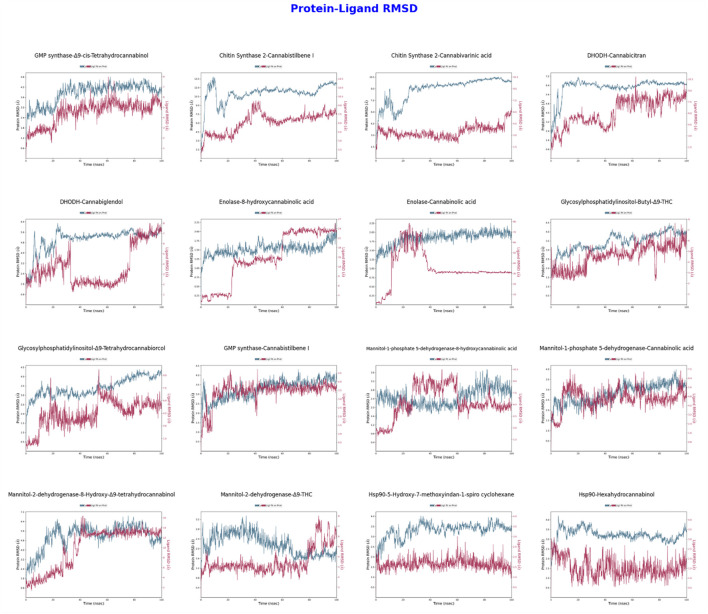
Protein-Ligand RMSD values obtained from molecular dynamics simulations conducted with two ligands for each of eight different proteins. The simulations were performed for 100 ns in the NPT ensemble at a temperature of 310 K. Each graph plots the RMSD values for both protein and ligand against time.

The RMSD analyses presented in Figure 3 have assessed the structural changes of protein-ligand complexes over time. In the GMP synthase-Δ9-cis-THC complex, the protein RMSD values remain steady between 2–4 Å, indicating structural stability, while the ligand RMSD values vary between 1–2 Å, suggesting a high binding stability of the ligand. Conversely, the CaChs2-Cannabistilbene I complex exhibits protein RMSD values fluctuating between 6–10 Å, indicating some flexibility in the protein structure. The ligand RMSD values, ranging from 4 to 7 Å, reflect movements within the ligand binding site. For the CaChs2-Cannabivarinic acid complex, the protein RMSD values are stable between 6–9 Å, while the ligand RMSD values range from 5 to 8 Å, indicating moderate ligand stability. In the DHODH-Cannabicitran complex, the protein RMSD values remain consistent between 1–2 Å, demonstrating a very stable structure, but the ligand RMSD values fluctuate significantly between 10–20 Å, indicating substantial movements within the ligand binding site. The Enolase-Cannabinolic acid complex displays protein RMSD values consistently between 1–2 Å, reflecting high structural stability, with ligand RMSD values between 2–6 Å, indicating good ligand stability. The M1P5DH-Cannabinolic acid complex exhibits protein RMSD values stable between 2–3 Å, indicating structural stability, with ligand RMSD values ranging from 2 to 4 Å, reflecting high ligand stability. The Mannitol-2-dehydrogenase-Δ9-THC complex displays protein RMSD values stable between 2–3 Å, indicating structural stability, with ligand RMSD values between 2–4 Å, reflecting high ligand stability. For the Hsp90-5-Hydroxy-7-methoxyindan-1-spiro cyclohexane complex, protein RMSD values remain steady between 2–3 Å, indicating structural stability, while ligand RMSD values range from 3 to 6 Å, reflecting moderate ligand stability. In the Hsp90-Hexahydrocannabinol complex, protein RMSD values are stable between 3–4 Å, indicating structural stability, while ligand RMSD values vary between 5–8 Å, indicating moderate ligand stability. This detailed analysis provides insights into the stability and flexibility of protein-ligand interactions across various complexes, highlighting the importance of both protein and ligand dynamics in the context of structural biology. The GMP synthase-Δ9-cis-THC, Enolase-Cannabinolic acid, M1P5DH-Cannabinolic acid, Mannitol-2-dehydrogenase-Δ9-THC, and Hsp90-5-Hydroxy-7-methoxyindan-1-spiro cyclohexane complexes stand out for their excellent performance, reflecting high stability and strong binding interactions, crucial factors for future structural or therapeutic considerations.

**FIGURE 4:**
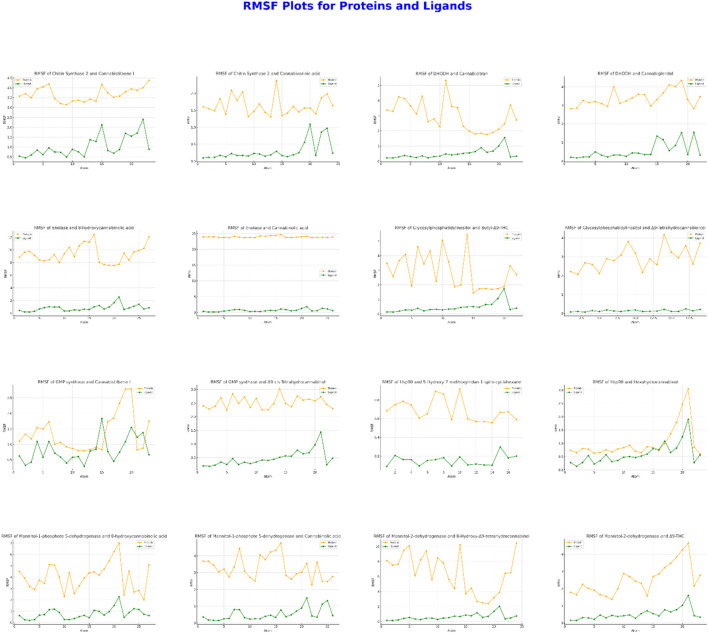
Protein-Ligand RMSF Values: Results Obtained from Molecular Dynamics Simulations with Two Ligands for Each of Eight Different Proteins. In Each Graph, the RMSF Values for Both Protein and Ligand are Plotted Against Atom Number.

The evaluations presented in Figure 4 are crucial for assessing these interactions. During the interaction between CaChs2 and Cannabidiolene I, high protein stability was observed, with slight increases in ligand RMSF (Root Mean Square Fluctuation) values at specific atoms. Conversely, the interaction with Cannabidiolic acid showed very low ligand RMSF values. In the interactions of DHODH protein with Cannabidiolic acid and Cannabidiolid, moderate protein mobility was noted, but the ligand RMSF values were lower compared to the protein. For Enolase, interactions with 9-Hydroxycannabidiolic acid exhibited increased mobility at certain atoms, whereas interactions with Cannabidiolic acid showed that the ligand stabilized the protein. The Glycosylphosphatidylinositol protein did not exhibit significant mobility differences in interactions with Bu-9-Δ9-THC and Δ9-Tetrahydrocannabidiolic acid. During the interaction of GMP Synthase with Cannabidiolene I, there was an increase in protein RMSF values, while there was no significant difference with cis-3-Hexenylcannabidiol. The Hsp90 protein displayed high mobility when interacting with 5-Hydroxy-7-methoxycoumarin-4-piperidone, whereas it exhibited low RMSF values with Hexahydrocannabinol. The M1P5DH protein showed low RMSF values and stability in interactions with both 8-Hydroxycannabidiolic acid and Cannabidiolic acid. However, in the interaction of Mannitol-2-dehydrogenase protein with 8-Hydroxy-Δ9-Tetrahydrocannabidiolic acid, a significant increase in RMSF was observed, particularly after atom 25, whereas no significant difference was noted with Δ9-THC. These results elucidate the impact of ligands on protein stability and reveal dynamic interactions in specific regions.

## 4 Discussion

This study aims to investigate the potential of selected cannabinoid and stilbenoid compounds as antifungal agents by evaluating their binding affinities and interaction profiles with eight fungal proteins. The eight fungal proteins investigated are associated with signaling pathways and biochemical functions that play critical roles in cellular homeostasis and pathogenic processes. Important information is included to uncover the antifungal potential of these targets by describing the molecular roles, biological mechanisms, and cellular locations of these proteins. The selected proteins include GPI, Enolase, M2DH, GMP Synthase, DHODH, Hsp90, Chitin Synthase, and M1P5DH. These proteins were included in the study because they contribute to a wide range of biological functions. In particular, GPI plays a central role in GPI anchor biosynthesis by regulating the binding of proteins to the cell surface and maintains the stability of the cellular structure (Álvarez-Sánchez et al., 2024). Therefore, GPI was selected as an antifungal target due to its potential to interfere with cell wall-associated biosynthetic processes. Enolase is involved in the basic pathways of energy metabolism such as glycolysis and ribonucleotide catabolism, and inhibition of these processes may prevent fungal growth by stopping energy production (Dasari et al., 2019; Díaz-Ramos et al., 2012). M2DH contributes to energy storage and osmotic balance by participating in the metabolism of mannitol and other polyol derivatives, which makes it an important strategic target that can weaken cellular stress resistance (Upadhyay et al., 2015). GMP Synthase plays a critical role in nucleotide biosynthesis pathways, especially ribonucleotide and purine biosynthesis, and inhibition of these processes can directly affect cell division (Oliver et al., 2013). DHODH supports *de novo* pyrimidine biosynthesis by localizing to the inner mitochondrial membrane, which is important for the production of nucleotides required for DNA and RNA synthesis (Hai et al., 2024; Rawls et al., 2000). Hsp90 regulates the resistance of pathogenic fungi to environmental stresses by acting in protein folding, stabilization and biological regulation processes, and therefore has significant potential as an antifungal target (Hoter et al., 2018; Wei et al., 2024). CaChs2 plays a critical role in cell wall biosynthesis through chitin synthesis and glycosyltransferase activities; since chitin is one of the main components of the fungal cell wall, inhibition of this protein may lead to weakening of the cell wall (Lenardon et al., 2010; Merzendorfer, 2011). M1P5DH is localized in the cytoplasm and contributes to energy storage and osmotic balance processes through mannitol metabolism; therefore, inhibition of mannitol may weaken the resistance of fungi to osmotic stress (Lim et al., 2021). Selecting proteins from different fungal strains increases the versatility and scope of our study. There are phylogenetic differences between these strains, and it is anticipated that proteins from each strain may exhibit unique properties in ligand binding performance. This approach has allowed us to better understand the diversity of ligand trafficking in proteins from different strains and to conduct comprehensive analyses in a wide biological spectrum. In addition, this diversity is crucial for increasing the potential of the selected ligands to work in a wide range of fungi as their biological targets, thereby enhancing the overall applicability of our findings. Furthermore, the multi-target nature of this study highlights the potential for synergistic effects by simultaneously inhibiting multiple fungal proteins involved in distinct yet complementary pathways. For instance, targeting proteins associated with energy metabolism, cell wall synthesis, and stress response simultaneously could enhance antifungal efficacy by disrupting multiple critical functions (Fisher et al., 2024).In our study, the selected protein targets were evaluated as protein models that could provide potential therapeutic effects on clinically important pathogenic fungal species. These protein targets are particularly associated with pathogenic species such as *Candida* albicans and Aspergillus fumigatus, which are the main causes of hospital-associated infections and can develop resistance to treatment. For example, Hsp90 plays a critical therapeutic role in important fungal pathogens such as *Candida* albicans, and this pathogen is known for its nosocomial infections and resistance to treatment. Aspergillus fumigatus, where the other target protein is GMP synthase, is another important pathogen that can become resistant to treatment and commonly cause infections in hospitals. In addition, proteins such as CaChs2 and Enolase AfEno1 are proteins that can potentially contribute to treatment by targeting the resistance mechanisms exhibited by pathogens such as *Candida* spp. and Aspergillus spp. against antifungal treatment. The selected protein targets are of greater clinical importance due to the resistance of fungal infections to treatment. For example, GPI is a critical component of the cell wall of *Candida* albicans, while enzymes such as M2DH and M1P5DH are part of critical metabolic pathways in Aspergillus fumigatus, and DHODH is an important biosynthetic enzyme in other fungal species such as Aspergillus nidulans. Selection of these proteins is directly linked to resistance to therapy in different fungal species, and inhibition of these proteins may allow the development of new therapeutic strategies.

In light of recent comprehensive reviews, such as (Rabaan et al., 2023; Fisher et al., 2022), which emphasize the increasing prevalence of antifungal resistance, the importance of multi-target strategies becomes even more apparent. Antifungal resistance mechanisms, including efflux pump overexpression, biofilm formation, and genetic alterations in target sites, significantly hinder the efficacy of current therapies. Addressing these mechanisms through a multi-target approach not only has the potential to mitigate resistance but also aligns with global initiatives aimed at developing novel antifungal agents with broader activity spectra and reduced resistance potential. While the potential for antagonistic interactions between targets must be carefully evaluated to optimize combination therapies, such strategies represent a promising avenue for enhancing treatment efficacy and overcoming the limitations of current antifungal agents.

Functional network analyses have revealed potential synergistic interactions and shared functions of these proteins in biological processes. For example, Enolase and GMP Synthase play similar roles in energy metabolism by participating in molecular functions such as “ATP binding”; Chitin Synthase and GPI share similar functions in maintaining cellular stability by participating in cell wall biosynthesis. Hsp90 and GMP Synthase are localized in the cytosol and plasma membrane, supporting cellular resistance mechanisms. These findings are important for our understanding of how proteins participate in various biological pathways and molecular functions and the coordination between them. By employing MD and MDS, ligands with the highest binding affinities were identified, and their pharmacokinetic profiles were analyzed using ADMET. The docking studies indicated that GMP synthase had the highest binding affinity with Cannabistilbene I (−9.1 kcal/mol), reflecting hydrophobic solid interactions and multiple hydrogen bonds. Similarly, CaChs2 demonstrated strong binding with Cannabistilbene I, suggesting its potential as an inhibitor. In contrast, ligands such as Cannabinolic acid and 8-hydroxycannabinolic acid exhibited moderate binding affinities with the target proteins, emphasizing the variability in interaction strengths among different compounds (Appendino et al., 2011; Kogan and Mechoulam, 2007).

ADMET analysis provided important information on the pharmacokinetic properties of the ligands. Hexahydrocannabinol and Δ9-cis-THC showed high lipophilicity and significant intestinal permeability; however, these ligands also carry the risk of HERG channel inhibition, which may limit their therapeutic applications. In contrast, ligands such as Cannabistilbene I and Cannabiglendol stand out as more suitable candidates, exhibiting lower risk of HERG inhibition and moderate lipophilicity. Solvent exposure analysis revealed significant differences in the binding environments of protein-ligand complexes. In particular, solvent exposure was observed for DHODH-Cannabicitran and GMP synthase-Δ9-cis-THC complexes, indicating potential changes in ligand stability and protein interactions in these environments (Jiang et al., 2019).

Consistent with the study by Mulia et al., ∆(9)-THC derivatives exhibited pharmacokinetic properties such as high lipophilicity (logP >5), intestinal permeability, and moderate brain penetration (Mulia et al., 2021). In our study, the calculated QPlogPo/w (5.663) and QPPCaco (4571.459) values for Δ9-cis-THC were found to be consistent with these results. The study of Thomas et al. examined the pharmacological properties of Δ(9)-THC analogs in more depth, and showed the effects of lipophilicity on a compound. In particular, it was stated that the addition of long side chains increased lipophilicity by approximately 3 times per CH2 group, and although no direct correlation with pharmacological activity was found, it was emphasized that lipophilicity plays a critical role in pharmacokinetic processes (Thomas et al., 1990). The QPlogPo/w value in our study (5.663) confirms the natural high lipophilicity of ∆(9)-THC, and as stated by Thomas et al., it also draws attention to the role of this lipophilicity in pharmacokinetic processes. However, the QPlogHERG value of Δ9-cis-THC (−4.894) indicates the risk of cardiotoxicity, complementing the findings of both Mulia and Thomas. Chetia et al., addressing the toxic effects of THC on brain dopamine levels and the attenuation of these effects with Cannabidiol, emphasized that THC affects the dopaminergic (DAergic) system in the brain, especially stimulating mesolimbic DA-containing neurons and increasing striatal dopamine levels. However, thanks to the anxiolytic and antipsychotic properties of CBD, it has been stated that THC has the potential to improve these variations on the DAergic system (Chetia and Borah, 2020). Δ9-cis-THC (Δ9-cis-THC) is an isomer that has attracted attention in low-THC Cannabis sativa cultivars, where it is found at levels comparable to Δ9-trans-THC (Jagannathan, 2020). This ligand was detected especially in low THC fiber hemp species but not in high THC medical cannabis varieties. This suggests that the natural abundance of Δ9-cis-THC may be related to genetic or environmental factors. It has been stated that Δ9-cis-THC has high enantiomeric purity (80%–90%) and this feature provides a significant advantage in the investigation of its biological effects (Schafroth et al., 2021). In our study, the selection of Δ9-cis-THC to evaluate its antifungal potential reveals the possible importance of both its natural abundance and chemical properties in interaction with target proteins. In particular, Δ9-cis-THC, which shows strong binding to targets such as GMP synthase, is considered a promising candidate for the development of new antifungal agents due to its potential to be easily obtained at low cost and its stability in biological systems. In this context, information on its natural abundance increases the importance of our study in terms of the sustainability and large-scale production of the compound.

The study by O'Croinin et al. addressing the pharmaceutical potential and predictive pharmacokinetic properties of stilbenes provides valuable data regarding the anti-inflammatory, antioxidant and anticancer effects of stilbenes, especially those derived from Cannabis sativa (O’Croinin et al., 2023). In the study, the pharmacokinetic profiles of Cannabistilbene I and other stilbenes were evaluated with parameters such as the potential to cross the BBB and the ability to pass through human skin and jejunal tissues. In this context, the high BBB permeability of Cannabistilbene I (84%) and its compliance with optimal Lipinski parameters indicate that the molecule is a promising candidate pharmacologically for neurological targets. In our study, ADMET analyses of Cannabistilbene I support its high lipophilicity and reliable bioavailability properties, and also emphasize the effects of the molecule especially on inflammatory mechanisms. The findings of both studies indicate that the physiochemical and pharmacokinetic properties of stilbenes increase the therapeutic potential, strengthening the consistency of the data in the current literature. These results reveal the need for further research to develop pharmaceutical formulations of stilbenes. The effect of Cannabistilbene I on Angiotensin II (Ang II)-induced cardiac hypertrophy was studied and how this effect was modulated by cytochrome P450 (CYP) enzymes and arachidonic acid (AA) metabolites. The findings indicate that Cannabistilbene I attenuates the cardiac hypertrophy-inducing effects of Ang II, reduces the increase in cellular surface area, and regulates the expression of hypertrophic marker genes. In addition, Cannabistilbene I was found to provide cardioprotective enzymatic activity by increasing CYP1A1 gene expression, increasing the levels of the metabolite 19(S)-HETE, and reversing the decline induced by Ang II (Alammari et al., 2024).

In our study, it was determined that stilbene derivatives such as Cannabistilbene I exhibit high lipophilicity and anti-inflammatory properties, and also show potential therapeutic effects in the cardiovascular system. In particular, ADMET analyses emphasized that Cannabistilbene I has BBB permeability, but its low lipophilic profile may reduce cardiotoxicity. The effects on Ang II-induced cardiac hypertrophy support the protective role of Cannabistilbene I against cardiovascular diseases and are consistent with our ADMET-based pharmacokinetic findings. In particular, stilbenes' effects on inflammation and cellular stress response were emphasized, demonstrating a cardioprotective potential at both molecular and cellular levels. In our study, the ADMET profile of Cannabidiolic Acid (CBDA) was evaluated in detail. Our findings regarding the pharmacokinetic and pharmacodynamic properties of CBDA largely overlap with the research conducted by Formato et al. Formato et al. In their study, the molecular weight (MW) of CBDA was determined as 358.48, the logP value measuring hydrophobicity was 6.43, the number of hydrogen bond acceptors (HBA) was 4 and the number of donors (HBD) was 3. In addition, the topological polar surface area (TPSA) of CBDA was calculated as 77.75 and it was determined that it contained 7 rotatable bonds (NRTOB). They emphasized that these parameters were positive in terms of oral bioavailability and showed that CBDA has a good absorption potential (Formato et al., 2020).

The antifungal activities and ADMET profiles of selected compounds such as Cannabistilbene I and Δ9-cis-THC were compared with those of the widely used clinical antifungal agents fluconazole (Kauthale et al., 2017) and amphotericin B (Khosravi et al., 2022) in this study. In the literature, it has been reported that the minimum inhibitory concentration (MIC) values of fluconazole against fungal pathogens such as *Candida* albicans, Aspergillus niger, and Aspergillus flavus are between 3.12 and 25 μg/mL, while amphotericin B, despite its high activity, has serious disadvantages such as nephrotoxicity and high cost. In our study, Cannabistilbene I exhibited high binding affinity with GMP Synthase (−9.1 kcal/mol) and CaChs2 (−9.1 kcal/mol), while Δ9-cis-THC showed a lower binding affinity with GMP Synthase (−7.2 kcal/mol). ADMET analyses show that Cannabistilbene I has safety properties similar to fluconazole with a low risk of cardiotoxicity and a high bioavailability profile. On the other hand, Δ9-cis-THC carries pharmacokinetic advantages such as high lipophilicity (QPlogPo/w = 5.663) and intestinal permeability (QPPCaco = 4571.459), while its higher hERG channel inhibition potential (−4.894) compared to fluconazole and amphotericin B poses a possible risk in terms of cardiotoxicity. While the efficacy of fluconazole is supported by MIC values, the antifungal activities of the compounds in our study were based on their binding affinities, which requires *in vitro* and *in vivo* validation to better understand their clinical potential. Cannabistilbene I and Δ9-cis-THC can be considered as innovative and potential candidates in antifungal therapy; However, comprehensive pharmacokinetic and toxicological studies as well as experimental confirmations are needed before it can be considered as an alternative to fluconazole and amphotericin B (Elias et al., 2022; Ghobadi et al., 2023; Martínez et al., 2024; Parveen and Balamurugan, 2023).

In our study, similarly strong oral bioavailability signals were found for CBDA and it was shown that the logP value was calculated as 6.2, TPSA as 79.1, HBA as 4, and HBD as 3. In addition, in our protein target analyses, CBDA’s G-protein coupled receptor (GPCR) binding score was evaluated as −0.41, ion channel modulator score as −0.06, kinase inhibitor score as −0.72, and nuclear receptor binding score as −0.28. These findings are very close to the values of −0.39, −0.05, −0.74, and −0.30 in the study by Formato et al. In addition, minimal deviations were observed in protease inhibitor and enzyme inhibitor activities, and the protease inhibitor score was found as −0.65 in our study and −0.63 in Formato et al. The pharmacokinetic profiles and antiseizure effects of phytocannabinoid acids (including CBDA) were examined in detail in the study by Lyndsey L. Anderson et al. (Anderson et al., 2019). In this study, it was reported that CBDA was rapidly absorbed and its brain-to-plasma ratio was low (≤0.04). However, when a different Tween 80-based carrier was used, this ratio increased to 1.9, emphasizing that this increased the brain penetration of CBDA. In addition, it was shown that CBDA delayed the occurrence of generalized tonic-clonic seizures by increasing the seizure threshold in mice with Dravet syndrome. Our study, on the other hand, examined the pharmacokinetic properties and biological activities of CBDA at the molecular level, including the evaluation of important pharmacokinetic parameters of CBDA such as QPlogPo/w, TPSA and NRTOB. The report of a low brain-to-plasma ratio by Anderson et al. and their emphasis on anti-seizure effects are parallel to our results confirming the pharmacokinetic potential of CBDA. In particular, the finding that the bioavailability and brain penetration of CBDA may vary depending on the carrier supports the relevance of our pharmacokinetic assessments for clinical applications. Current studies on cannabicitran have indicated that this compound is often found at levels of up to 10% in commercial “purified” CBD extracts. The literature suggests that cannabicitran is racemic in nature and may be formed during herbal extraction processes (Wood et al., 2023). In contrast, the evaluations made on the pharmacokinetic profile of cannabicitran in our study provided new information, especially on its structural and pharmacological properties. ADMET results obtained from the literature provide critical information regarding the bioavailability of cannabicitran and its interaction with target receptors. For example, it is known that this compound has affinity for receptors such as 5-HT1A. While our study provides preliminary information for a better understanding of the metabolic and biological effects of cannabicitran, it can be expanded to provide full compliance with the literature with more comprehensive ADMET analyses and abundance data. In this context, in-depth studies are required at both the biosynthesis and biological effects levels to evaluate the pharmacological potential of cannabicitran.

The analyses made on CBDVA in our study evaluated the pharmacokinetic properties and potential anticonvulsant effects of the compound. In our study, CBDVA significantly increased the thermal seizure threshold at certain doses (e.g., 100 mg kg⁻^1^), indicating the antiseizure effect of the compound. These findings support a study by Anderson et al. (2021). In the study by Anderson et al., it was reported that CBDVA, as well as other phytocannabinoid acids such as CBGA and CBGVA, exhibited protective effects against thermal seizures in the Dravet syndrome mouse model. In particular, this study showed that CBDVA had a neuroprotective effect against thermal seizures after acute intraperitoneal administration, but Δ9-THCV (at a dose of 3 mg kg⁻^1^) had a proconvulsant effect in contrast. Our study suggests that CBDVA may be a promising agent in the treatment of epileptic diseases such as Dravet syndrome, and supports the fact that this compound should be considered in future drug development processes for epilepsy, together with the results of Anderson et al. Among the 16 ligands evaluated in our study, considering that certain ligands may be higher in natural abundance than others, this situation stands out as an important finding when considered together with RMSD and RMSF analyses. In particular, ligands exhibiting high RMSF stability and low RMSD values supported strong interactions with the target protein. These ligands, which are naturally more abundant, offer the potential to improve both their availability in biological systems and their pharmacokinetic profiles. Considering this relationship, the findings supported by protein-ligand contact and ADMET predictions suggest that naturally more abundant compounds may be more pharmacologically suitable candidates. For example, although the natural abundances of ligands such as cannabicitran have been reported in the literature, in this study they were found to support this assumption with their strong stability and interaction profiles. These findings demonstrate that our study is not only relevant to pharmacological profile assessments, but also to the effect of ligand abundance on therapeutic potential. RMSD analyses are crucial for evaluating the structural stability of protein-ligand complexes over time. In the GMP synthase-Δ9-cis-Tetrahydrocannabinol (Δ9-cis-THC) complex, the protein RMSD values remain stable between 2–4 Å, indicating the structural stability of the protein-ligand interaction. This suggests that the ligand binds firmly and stably to the protein. On the other hand, in the CaChs2-Cannabistilbene I complex, the protein RMSD values fluctuate between 6–10 Å, indicating some flexibility in the protein structure. This flexibility allows more movement in the ligand binding site, leading to higher ligand RMSD values (4–7 Å). In the DHODH-Cannabicitran complex, the protein RMSD values remain steady between 1–2 Å, demonstrating high structural stability; however, the ligand RMSD values fluctuate widely between 10–20 Å, indicating significant movements within the ligand binding site, which might be related to solvent exposure.

RMSF analyses are critical for evaluating protein-ligand interactions' dynamic flexibility and stability. During the interaction between CaChs2 and Cannabistilbene I, high protein stability was observed, with slight increases in ligand RMSF values at specific atoms. In contrast, the interaction with Cannabinolic acid showed very low ligand RMSF values, indicating that Cannabinolic acid stabilizes CaChs2, resulting in less dynamic flexibility in the protein-ligand interaction. In the interactions of DHODH protein with Cannabinolic acid and Cannabiglendol, moderate protein mobility was observed, but the ligand RMSF values were lower compared to the protein. These results highlight the impact of ligands on protein stability and reveal dynamic interactions in specific regions.

For Enolase, interactions with 8-hydroxycannabinolic acid showed increased mobility at certain atoms, whereas interactions with Cannabinolic acid indicated that the ligand stabilized the protein. The Glycosylphosphatidylinositol protein did not show significant mobility differences in interactions with 3-Butyl-Δ9-THC and Δ9-Tetrahydrocannabinorcol. During the interaction of GMP synthase with Cannabistilbene I, an increase in protein RMSF values was observed, while no significant difference was noted with Δ9-cis-THC. The Hsp90 protein displayed high mobility when interacting with 5-Hydroxy-7-methoxyindan-1-spiro cyclohexane, whereas it exhibited low RMSF values with Hexahydrocannabinol. The M1P5DH protein showed low RMSF values and stability in interactions with both 8-hydroxycannabinolic acid and Cannabinolic acid. However, in the interaction of Mannitol-2-dehydrogenase protein with 8-Hydroxy-Δ9-Tetrahydrocannabinol, a significant increase in RMSF was observed, particularly after atom 25, whereas no significant difference was noted with Δ9-THC. These results elucidate the impact of ligands on protein stability and reveal dynamic interactions in specific regions.

The differences between the binding affinities obtained in our study are due to the diversity in the chemical and structural properties of the protein targets and ligands. In particular, the hydrogen bonding potentials, hydrophobic surface areas and electrostatic interactions of the ligands are among the determining factors of these differences (Chen et al., 2016; Patil et al., 2010). Differences in the active sites of protein targets may also contribute to the changes observed in ligand binding affinities. For example, while extensive hydrogen bonding networks in proteins such as Hsp90 lead to high binding affinities, hydrophobic regions in targets such as GMP Synthase may be effective in ligand selection (Hoxie and Street, 2020). In similar studies in the literature, studies comparing the binding properties of different ligands to the same protein targets show that the changes in binding affinities are affected by the chemical modifications of the ligands or the conformational flexibility of the protein targets (Ferenczy and Kellermayer, 2022; Madushanka et al., 2023; Patil et al., 2010). In future studies, detailed examinations are planned with methods such as molecular dynamics simulations and energy component analysis of ligand-protein complexes to better understand the differences in binding affinities. This approach may contribute to the design of structural modifications that will increase the binding affinities of ligands.

The choice to employ MD and MDS in this study is well-founded due to their complementary nature in evaluating protein-ligand interactions. Molecular docking serves as an initial screening tool, allowing for the identification of the most promising ligand candidates based on binding affinities (Agu et al., 2023; Salmaso and Moro, 2018). However, docking alone cannot fully capture these interactions' dynamic and temporal aspects, which are crucial for understanding the stability and behavior of protein-ligand complexes in a physiological environment. Therefore, MDS was utilized to provide a more comprehensive assessment of the binding stability and dynamic interactions over time (Shukla and Tripathi, 2021). Furthermore, the decision to utilize a 100 ns simulation duration for MDS was based on a balance between computational feasibility and the ability to capture significant conformational changes and stabilization patterns within the protein-ligand complexes (Koshy et al., 2010). While longer simulations could potentially provide additional insights, previous studies have demonstrated that a 100 ns timeframe is sufficient to observe key interaction features, stabilize RMSD values, and gain meaningful insights into protein flexibility and ligand binding modes. Therefore, this duration was considered appropriate for effectively investigating the dynamic behaviors of the complexes and their potential antifungal properties (Al-Karmalawy et al., 2021). The combination of MD followed by MDS ensured a robust evaluation of the selected ligands’ potential as antifungal agents, supporting their future experimental exploration as promising drug candidates.

This study provides a comprehensive analysis of interactions between various fungal proteins and ligands, revealing the antifungal potential of selected compounds. Cannabistilbene I exhibited strong binding affinities (−9.1 kcal/mol) and stable dynamic profiles with GMP Synthase and CaChs2, while ADMET analyses highlighted its high bioavailability and low cardiotoxicity risk. Similarly, Δ9-cis-THC stood out as another promising candidate due to its natural abundance and protein-ligand stability. These findings suggest that both molecules are promising candidates for antifungal drug development and underscore the importance of further experimental validation.

## 5 Conclusion

This study provides a comprehensive assessment of how selected cannabinoid and stilbenoid compounds interact with eight different fungal proteins, highlighting the promising potential of these compounds as antifungal agents. Through MD simulations and analyses, ligands with high binding affinity, particularly interacting with GMP Synthase and CaChs2, were identified. These ligands stand out as strong candidates as potential inhibitors. In particular, Cannabistilbene I exhibited exceptional binding affinity with both GMP Synthase (−9.1 kcal/mol) and CaChs2, supported by stable RMSD values and significant hydrogen bond interactions. Furthermore, Δ9-cis-THC exhibited low RMSD values (2–4 Å) and strong hydrophobic interactions with GMP Synthase, demonstrating its potential as a potent initiator compound for antifungal applications. ADMET analysis reinforced these findings, revealing that Cannabistilbene I presented favorable pharmacokinetic profiles such as low risk of HERG inhibition and moderate lipophilicity, while Δ9-cis-THC showed a mild risk of cardiotoxicity despite high intestinal permeability and promising bioavailability. One of the major strengths of this study is the detailed establishment of interaction profiles between different proteins and ligands. This broad scope helps us to understand the biological activities and antifungal capacities of these compounds in more depth. The study showed that stable protein-ligand interactions with low RMSD and RMSF values are important indicators of antifungal potential. However, it should be noted that these promising *in silico* results need to be confirmed by *in vitro* and *in vivo* experiments to fully confirm the efficacy of these compounds. Future research should prioritize the experimental evaluation and optimization of lead candidates, particularly Cannabistilbene I and Δ9-cis-THC, to better understand the validity and efficacy of these compounds as clinical antifungal agents. In conclusion, this study highlights the therapeutic potential of cannabinoids and stilbenoids and provides a solid foundation for the development of new antifungal therapies.

## Data Availability

The original contributions presented in the study are included in the article/Supplementary Material, further inquiries can be directed to the corresponding authors.
