# Supplementary material for: Exploring the antifungal potential of Cannabis sativa-derived stilbenoids and cannabinoids against novel targets through in silico protein interaction profiling
Source: Front Chem. 2025 Jan 6;12:1515424. doi: 10.3389/fchem.2024.1515424 (PMC11743709; doi:10.3389/fchem.2024.1515424)
Supplement: Supplementary file 1 [file Table1.docx]

Supplementary Material

# Supplementary Data1

**Cannabinoid Compounds (Continued)**

1. Cannabinodivarin (C19H22O2)
2. Cannabivarin (C19H22O2)
3. Δ9-trans-Tetrahydrocannabivarin (C19H26O2)
4. Cannabichromevarin (C19H26O2)
5. Cannabicyclovarin (C19H26O2)
6. Cannabidivarin (C19H26O2)
7. Δ9-cis-Tetrahydrocannabivarol (C19H26O2)
8. (+)-Δ7-cis-Isotetrahydrocannabivarin (C19H26O2)
9. (-)-Δ7-cis-Isotetrahydrocannabivarin (C19H26O2)
10. (-)-Δ7-trans-Isotetrahydrocannabivarin (C19H26O2)
11. Bis-norcannabielsoin (C19H26O3)
12. Cannabigerovarin (C19H28O2)
13. Cannabiglendol (C19H28O3)
14. Nor-Cannabinol (cannabinol-C4) (C20H24O2)
15. Cannabichromevarinic acid (C20H26O4)
16. Cannabidivarinic acid (C20H26O4)
17. D9-trans-Tetrahydrocannabivarinic acid A (C20H26O4)
18. Bis-norcannabielsoic acid B (C20H26O5)
19. Cannabidibutol (norcannabidiol) (C20H28O2)
20. Δ9-trans-nor-Tetrahydrocannabinol (butyl-D9-tetrahydrocannabinol) (C20H28O2)
21. Cannabigerovarinic acid (C20H28O4)
22. Cannabioxepane (C21H22O2)
23. Dehydrocannabifuran (C21H24O2)
24. Cannabinol (C21H26O2)
25. Cannabinodiol (C21H26O2)
26. Cannabifuran (C21H26O2)
27. 8-Hydroxcannabinol (C21H26O3)
28. (1’S)-Hydroxycannabinol (C21H26O3)
29. (-)-Δ7-trans-Isotetrahydrocannabinol (C21H28O2)
30. Dihydrocannabinol (C21H28O2)
31. 8-Oxo-Δ9-trans-tetrahydrocannabinol (C21H28O3)
32. Δ9-trans-nor-Tetrahydrocannabinolic acid (C21H28O4)
33. 10αR-Hydroxy-10-oxo-Δ8-trans-tetrahydrocannabinol (C21H28O4)
34. Cannabichromene (C21H30O2)
35. Cannabicyclol (cannabipinol) (C21H30O2)
36. Cannabidiol (C21H30O2)
37. Δ9-trans-Tetrahydrocannabinol (C21H30O2)
38. Δ9-cis-Tetrahydrocannabinol (C21H30O2)
39. Δ8-trans-Tetrahydrocannabinol (C21H30O2)
40. Cannabicitran (C21H30O2)
41. 8a-Hydroxy-Δ9-trans-tetrahydrocannabinol (C21H30O3)
42. 10α-Hydroxy-Δ8-trans-tetrahydrocannabinol (C21H30O3)
43. 2-Geranyl-5-hydroxy-3-n-pentyl-1,4-benzoquinone (C21H30O3)
44. (±)-3‘‘-Hydroxy-Δ(4’’,5‘‘)-cannabichromene (C21H30O3)
45. Cannabielsoin (C21H30O3)
46. 8b-Hydroxy-Δ9-trans-tetrahydrocannabinol (C21H30O3)
47. 10β-Hydroxy-Δ8-trans-tetrahydrocannabinol (C21H30O3)
48. 9β,10β-Epoxyhexahydrocannabinol (C21H30O3)
49. 10α-Hydroxy-D9,11-hexahydrocannabinol (C21H30O3)
50. Cannabimovone (C21H30O4)
51. 7-Oxo-9α-hydroxyhexahydrocannabinol (C21H30O4)
52. (-)-Cannabitetrol (C21H30O5)
53. Cannabigerol (C21H32O2)
54. Cannabinerol (C21H32O2)
55. Hexahydrocannabinol (C21H32O2)
56. (-)-7-Hydroxycannabichromane (C21H32O3)
57. 9α-Hydroxyhexahydrocannabinol (C21H32O3)
58. 10α-Hydroxyhexahydrocannabinol (C21H32O3)
59. 10β-Hydroxyhexahydrocannabinol (C21H32O3)
60. 10αR-Hydroxyhexahydrocannabinol (C21H32O3)
61. Cannabiripsol (9,10-dihydroxyhexahydrocannabinol) (C21H32O4)
62. (+)-6,7-cis-Epoxycannabigerol (C21H33O3)
63. (-)-6,7-cis-Epoxycannabigerol (C21H33O3)
64. (+)-6,7-trans-Epoxycannabigerol (C21H33O3)
65. (-)-6,7-trans-Epoxycannabigerol (C21H33O3)
66. Carmagerol (C21H34O4)
67. Cannabinolic acid (C22H26O4)
68. 8-Hydroxycannabinolic acid (C22H26O5)
69. Cannabinol monomethyl ether (O-methylcannabinol) (C22H28O2)
70. Δ9-trans-Tetrahydrocannabinol aldehyde (C22H30O3)
71. Δ9-trans-Tetrahydrocannabinolic acid A (C22H30O4)
72. Δ8-trans-Tetrahydrocannabinolic acid (C22H30O4)
73. Cannabichromenic acid (C22H30O4)
74. Cannabicyclolic acid (C22H30O4)
75. Cannabidiolic acid (C22H30O4)
76. Δ9-trans-Tetrahydrocannabinolic acid B (C22H30O4)
77. Cannabielsoic acid B (C22H30O5)
78. Cannabielsoic acid A (C22H30O5)
79. Cannabidihexol (C22H32O2)
80. Cannabidiol monomethyl ether (C22H32O2)
81. Δ9-Tetrahydrocannabihexol (C22H32O2)
82. Cannabigerolic acid (C22H32O4)
83. Cannabinerolic acid (C22H32O4)
84. Cannabigerol monomethyl ether (C22H34O2)
85. 5-Acetoxy-6-geranyl-3-n-pentyl-1,4-benzoquinone (C23H32O4)
86. (±)-4-Acetoxycannabichromene (C23H32O4)
87. (-)-trans-Cannabidiphorol (C23H34O2)
88. (-)-Δ9-trans-Tetrahydrocannabiphorol (C23H34O2)
89. Cannabigerolic acid monomethyl ether (C23H34O4)
90. 5-Acetyl-4-hydroxycannabigerol (C23H35O4)
91. (+)-6,7-cis-Epoxycannabigerolic acid (C23H35O4)
92. (-)-6,7-cis-Epoxycannabigerolic acid (C23H35O4)
93. (+)-6,7-trans-Epoxycannabigerolic acid (C23H35O4)
94. (-)-6,7-trans-Epoxycannabigerolic acid (C23H35O4)
95. 4-Acetoxy-2-geranyl-5-hydroxy-3-n-pentylphenol (C23H35O4)
96. 11-Acetoxy-Δ9-trans-tetrahydrocannabinolic acid A (C24H32O6)
97. Sesquicannabigerol (C26H40O2)
98. 4-Terpenyl cannabinolate (C32H42O4)
99. epi-Bornyl Δ9-trans-tetrahydrocannabinolate (C32H45O4)
100. Bornyl Δ9-trans-tetrahydrocannabinolate (C32H45O4)
101. α-Terpenyl Δ9-trans-tetrahydrocannabinolate (C32H45O4)
102. 4-Terpenyl Δ9-trans-tetrahydrocannabinolate (C32H45O4)
103. β-Fenchyl Δ9-trans-tetrahydrocannabinolate (C32H47O4)
104. α-Fenchyl Δ9-trans-tetrahydrocannabinolate (C32H47O4)
105. ɣ-Eudesmyl Δ9-trans-tetrahydrocannabinolate (C37H53O4)
106. α-Cadinyl Δ9-trans-tetrahydrocannabinolate (C37H53O4)
107. ɣ-Eudesmylcannabigerolic acid (C37H55O4)
108. α-Cadinylcannabigerolic acid (C37H55O4)
109. Cannabidiolic acid tetrahydrocannabitriol ester (C43H58O7)
110. Cannabisol (C43H60O4)

**Supplementary Data: Stilbenoid Compounds (Continued)**

1. β-cannabispiranol (C15H20O3)
2. α-cannabispiranol (C15H20O3)
3. Acetyl cannabispirol (C17H22O4)
4. 5,7-dihydroxyindan-1-spirocyclohexane (C15H20O2)
5. 7-hydroxy-5-methoxyindan-1-spiro-cyclohexane (C15H20O3)
6. 5-hydroxy-7-methoxyindan-1-spiro-cyclohexane (C15H20O4)
7. Canniprene (C21H26O4)
8. Cannabistilbene I (C20H24O3)
9. Cannabistilbene IIa (C17H20O5)
10. Dihydroresveratrol (C14H14O3)
11. 9,10-dihydrophenanthrene (C14H12)
12. Cannithrene-1 (C15H14O3)
13. Cannithrene-2 (C16H16O4)
14. 4,7-dimethoxy-1,2,5-trihydroxyphenanthrene (C16H14O5)
15. 4,5-dihydroxy-2,3,6-trimethoxy-9,10-dihydrophenanthrene (C17H18O5)
16. 4-hydroxy-2,3,6,7-tetramethoxy-9,10-dihydrophenanthrene (C18H20O5)
17. Denbinobin (5-hydroxy-3,7-dimethoxy-1,4-phenanthrenequinone) (C16H12O5)
18. 3,40-dihydroxy-5,30-dimethoxy-50isoprenyl
19. Cannabistilbene IIb
20. 3,4-dihydroxy-5-methoxybibenzyl
21. 3,30-dihydroxy-5,40-dimethoxybibenzyl
22. 3,30-dihydroxy-5,40-dimethoxybibenzyl
